# LncRNA-42060 Regulates Tamoxifen Sensitivity and Tumor Development via Regulating the miR-204-5p/SOX4 Axis in Canine Mammary Gland Tumor Cells

**DOI:** 10.3389/fvets.2021.654694

**Published:** 2021-06-21

**Authors:** Enshuang Xu, Mengxin Hu, Reidong Ge, Danning Tong, Yuying Fan, Xiaoli Ren, Yun Liu

**Affiliations:** ^1^Department of Veterinary Surgery, College of Veterinary Medicine, Northeast Agricultural University, Key Laboratory of Comparative Medicine, Harbin, China; ^2^College of Veterinary Medicine, Henan University of Animal Husbandry and Economy, Zhengzhou, China

**Keywords:** canine mammary gland tumor, tamoxifen-resistant, lncRNA, miR-204-5p, Sox4

## Abstract

Tamoxifen is the drug of choice for endocrine therapy of breast cancer. Its clinical use is limited by the development of drug resistance. There is increasing evidence that long non-coding RNAs (lncRNAs) are associated with tumor drug resistance. Therefore, we established two TAM-resistant cell lines, CHMp^TAM^ and CHMm^TAM^. The different expression levels of lncRNA and miRNA in CHMm^TAM^ and CHMm were screened by RNA sequencing, and the lncRNA-miRNA interactions were analyzed. LncRNA ENSCAFG42060 (lnc-42060) was found to be significantly upregulated in drug-resistant cells and tumor tissues. Further functional validation revealed that the knockdown of lnc-42060 inhibited proliferation, migration, clone formation, restoration of TAM sensitivity, and reduction of stem cell formation in drug-resistant cells, whereas overexpression of lnc-4206 showed opposite results. Bioinformatics and dual-luciferase reporter gene assays confirmed that lnc-42060 could act as a sponge for miR-204-5p, further regulating SOX4 expression activity and thus influencing tumor cell progression. In conclusion, we screened lncRNAs and miRNAs associated with TAM resistance in canine mammary gland tumor cells for the first time. lnc-42060 served as a novel marker that may be used as an important biomarker for future diagnosis and treatment.

## Introduction

For Original Research Articles, Clinical Trial Articles, and Technology Reports the introduction should be succinct, with no subheadings. For Case Reports the Introduction should include symptoms at presentation, physical exams, and lab results. Breast cancer (BC) is a highly heterogeneous cancer. According to GLOBOCAN, about 2.1 million women were diagnosed with BC between 2018 and 2019, maked it the second-highest mortality disease among women. According to the expression of estrogen receptor (ER), progesterone receptor (PR), and human epidermal growth factor 2 (Her-2), the four main subtypes of BC are luminal A (ER+ and/or PR+, Her-2-), luminal B (ER+ and/or PR+, Her-2+), Her-2 overexpressing (ER- and PR-, Her-2+), and triple-negative breast cancer (TNBC; ER-, PR-, and Her-2-). Most BC patients belong to the luminal subtype (1). Different staging methods correspond to different treatment options for BC. Currently, endocrine therapy or anti-Her-2 targeted therapy in combination with chemotherapy is the mainstay of treatment. New clinical studies have shown that molecularly targeted therapy combined with endocrine therapy can improve progression-free survival in some BC patients, such as the mTOR inhibitor and the cyclin-dependent kinase 4/6 (CDK 4/6) inhibitor (2). Tamoxifen (TAM) is the drug of choice for endocrine therapy, not only as a therapeutic agent resulting in a 25–30% reduction in mortality, but also for the prevention of BC (3). However, with increasing drug use, scholars have found that in addition to some patients with primary resistance being themselves insensitive to TAM, about 30% of the women who are chronically exposed to the drug develop new drug resistance and are at risk of relapse within the next 10 years. This has resulted in the attenuation of endocrine therapy (4). Scholarly in-depth studies have suggested several possible factors that explain TAM resistance (TAMR), such as molecular pathways (ER, PI3K, RTKS signaling pathway), androgen receptor (AR) expression, HER-2 expression, G protein-coupled estrogen receptor (GPER), and non-coding RNA (ncRNA) (5). The mechanisms of TAMR involve numerous relevant factors, and most of them have not been elucidated clearly. Therefore, extensive research on BC treatment resistance is urgently needed to develop new predictive biomarkers and therapeutic targets for treatment.

Long non-coding RNAs (lncRNAs) are over 200 nt in length. They can be transcribed from a variety of genomic locations, such as at the promoter, enhancer, intron, antisense coding region of a gene, or independently in the genome. They have been shown to function as transcriptional signals, decoys, scaffolds, directing enhancer RNAs, and short peptides. In cancer, lncRNAs function through multiple mechanisms, such as chromatin remodeling, chromatin interactions, competing endogenous RNAs (ceRNAs), and natural antisense transcripts (6). Shen et al. (7) found 1,758 altered lncRNAs in BC. In addition, Yang et al. (8) found that more than 1,300 lncRNAs were differentially expressed in HER2+ BC. These results emphasize the relevant role of lncRNAs in the development of BC. TamR is one of the major therapeutic problems in BC. Therefore, lncRNAs modulate endocrine therapy resistance and may be a key factor in the treatment of resistance to endocrine therapy. Mechanistic studies to evaluate the role of lncRNAs in TamR have only emerged in the last few years. LncRNA UCA1 is the most studied lncRNA. It has been shown that exosomes from TAM-resistant cells (TAMRs) are enriched in UCA1 and can induce resistance in other sensitive cells (9). In addition, BCAR4, HOTAIR, CCAT2, DSCAM AS1, UC.57, GAS5 Linc00894-002, H19, and MALAT1 (10, 11). Although some lncRNAs have been studied, it has been gradually elucidated that some lncRNAs play an important role in a variety of biological phenomena. However, the expression of lncRNAs tends to be highly cell-and tissue-specific. At the same time, compared to tens of thousands of lncRNAs, our understanding of them is only the tip of the iceberg. Therefore, it is important to screen more lncRNAs and investigate their functions in the development of cancer. In particular, TAM-associated lncRNAs have been reported for the first time in animals.

Epithelial-mesenchymal transition (EMT) is defined as a biological process (BP) in the malignant pathogenesis of cancer cell reprogramming from an epithelial state to a mesenchymal state. This transition is closely associated with tumor proliferation, metastasis, drug resistance, apoptosis, and population diversity. Cancer stem cells (CSC) consist of cancer cells with stem-like features that have the ability to self-renew and differentiate. This differentiation and de-differentiation are closely related to EMT. Some authors have shown that the metastatic ability of CSC with EMT properties is enhanced, which led to the concept of metastatic stem cells (12, 13). This is mainly due to the loss of tight junctions and cell adhesion after acquiring the EMT phenotype, which prepares the cells for migration to new organs. This illustrates the inextricable link between EMT and CSC. Importantly, the potential link between EMT and CSC is a key to the acquisition of cancer drug resistance as well as cancer cell plasticity, in which cancer cells transform into malignant cells and vice versa (14). Therefore, in order to understand the mechanisms of cancer drug resistance, the characteristics of EMT and CSC should be determined. The migration ability of cells often predicts changes in EMT characteristics, while the formation of stem cell microemulsion spheres represents CSCs.

In summary, canine mammary gland tumor (CMGT) TamR cell lines CHMm^TAM^ and parent cell line CHMm were used for high-throughput sequencing to detect changes in the expression profiles of lncRNAs and miRNAs as well as to construct their regulatory networks. A new lncRNA-ENSCAFG42060 (lnc-42060) encoding on chromosome 25 (chr25: 9,055,794-9,074,347) was found to be overexpressed in CHMm^TAM^ for the first time. In addition, we tested its function in different cell types. The results show that lnc-42060 can act as a competitive endogenous RNA (ceRNA) that adsorbs miR-204-5p and promotes TamR, metastasis, EMT, and CSC processes through the SOX4 signaling pathway. This study is the first to report on TamR-associated ncRNAs in canines, and also validates the function of novel lnc-42060. The search for new biomarkers can help restore drug sensitivity and improve patient survival.

## Materials and Methods

### Clinical Subjects and Specimens

A total of 24 cases of CMGT specimens were recruited at Northeast Agricultural University Animal Hospital. The tissue was identified and classified (15). All ingredients are permitted by pet owners and are in accordance with the Experimental Animal Control law of Northeast Agricultural University. Animal care and use procedures are approved by the Ethics Committee of Animal Hospital of Northeast Agricultural University.

### Cell Lines and Cell Culture

CHMp and CHMm cell line derived from CMGT, isolated from the primary and metastatic lesions (pleural effusion) of a 12-year-old female mutt with tumor, and presented by the department of Veterinary Medical Sciences, University of Tokyo, Japan (16). HEK293T was obtained from ATCC. TAMR CMGT cell lines CHMp^TAM^/CHMm^TAM^, collectively called TAMRs, were constructed by drug concentration gradient plus drug maintenance method (The final concentration of TAM is 10 μm) for 10 months. The cells were cultured to contain phenol-red-free DMEM medium (Invitrogen, Carlsbad, Calif, USA, SH30284) containing 10% charcoalstripped, steroid-depleted FBS (Sigma, USA, F6765), antibiotics-amphotericin (Beyotime Biotechnology, ShangHai, China, C0223), and 4-hydroxytamoxifen (Sigma, H7904).

### Transcriptome Sequencing

Three replicates of total RNA were extracted from CHMm^TAM^ and CHMm cell lines using a high-throughput microarray assay to screen for differentially expressed lncRNAs and miRNAs, respectively. After library construction and quality control, HiSeq sequencing platform was used for detection (NovelBio, Shanghai, China).

### Cell Transfection

The miR-204-5p NC, mimic, inhibitor, lnc-42060 siRNA and SOX4 siRNA were purchased from Ribiobio. The lnc-42060 sequence was sub-cloned into the pcDNA3.1 vector (Invitrogen, Carlsbad, Calif, USA) to enhance lnc-42060 expression (Bioengineer, Shanghai, China). The transfection was performed using Lipofectamine 2000 (Invitrogen, 11668019) according to the manufacturer's instructions.

### Transwell Migration Assay

1 × 10^5^ cells drug-treated cells were diluted in serum-free medium and seed on the upper compartment of the chamber (Corning, USA). In the lower chamber, 600 μL of consistent complete medium containing 20% fetal bovine serum was added and returned to the incubator for 24 h. Then the under chamber cells were fixed with 4% paraformaldehyde for 20 min, stained with 0.5% crystal violet dye for 10 min, rinsed with distilled water 2–3 times, and dried in a fume hood. Cells placed on transparent plates were photographed under an inverted microscope (200×). At least 3 fields were randomly selected from each group of cell samples and counted.

### Clone Formation Assay

Five hundred cells/well were seeded in 6-well culture plates. Replace the fresh culture medium on the fifth day. On the tenth day after washing with PBS, 4% paraformaldehyde was added and fixed for 15 min, and then crystalline violet staining was added.

### Tumor Sphere Formation Assays

A mixture culture medium was prepared and included serum-free F12 medium (Invitrogen, 21127022), 2% B-27 (Invitrogen, A3695201), 20 ng/mL basal fibroblast growth factor (bFGF) (Invitrogen, PHG0360), 20 ng/mL epidermal growth factor (EGF) (Invitrogen, RP-8661). 10^4^ cells were inoculated in 6-well ultralow attachment plates. After 14 days of culture, the diameter >75 μm was counted as a microemulsion sphere (17).

### Reverse-Transcription Quantitative PCR (QRT-PCR)

Total RNA was isolated from all cells using TRIZOL reagent (Invitrogen, 15596026). The PrimeScript RT reagent Kit (Takara, Shiga, Japan, RR047A) was used when the product was mRNA or lncRNA; When the inversion product is miRNA, the miScript Reverse Transcription Kit (Tiangen Biotech, Shangai, China, KR211) is used, and the specific operation is referred to the product instructions. The SYBR Premix Ex Taq™ Kit (TaKaRa, RR820A) was used for PCR testing. The internal parameters of mRNA or lncRNA were GAPDH, miRNA is U6. Finally, Light Cycler®480 System (Roche, Basel, Switzerland) was used for quantitative analysis. In this experiment, each gene of each cell sample was detected at least 3 times, and the mean value was calculated. The experimental data were analyzed by 2-ΔΔCT method. The primer sequence is shown in Supplementary Table 1.

### Nucleus-Cytoplasmic Separation

The Ambion® PARIS™ system (Invitrogen, AM1921) was used for the separation of nuclear and cytoplasmic fractions from TAMRs, according to the kit instructions.

### Double Luciferase Reporter Assay

lnc-42060 and 3′ untranslated region (3′ UTR) of SOX4 (Bioengineer, Shanghai, China) containing the miR-204 binding sites and corresponding mutated sequences (Supplementary Table 2) were synthesized and individually cloned into wild type (pMIR-REPORT vector-WT) and mutant type (pMIR-REPORT vector-MT), and named pMIR-WT or pMIR-MT. 293T cells were co-transfected with pMIR-WT or pMIR-MT, miR-204 mimic (Ribiobio, Guangzhou, China), and phRL-TK vector using Lipofectamine 2000 reagent. The DualGLO® Luciferase Assay System (Promega, USA, E1910 and E1960) was used to determine the final results.

### RNA Immunoprecipitation (RIP) Assay

RIP assays were performed using the RNA Immunoprecipitation Kit (Geneseed, Guangzhou, China, P0101) following the manufacture, rs protocol. Briefly, the cells were lysed with RIP lysis buffer and then Cell extracts are incubated with RIP buffer containing magnetic beads bound to human anti-ago2 antibody (Millipore) and NC normal mouse IgG (Millipore). Samples were then incubated with Proteinase K buffer with shaking. Finally, the RNAs in the immunoprecipitates were extracted, purified, and subjected to real-time fluorescence quantitative PCR.

### Western Blot

Cellular proteins were extracted by the BCA kit (Beyotime Biotechnology, P0012S). The samples were subjected to SDS-polyacrylamide gel electrophoresis to separate the target proteins. Separated proteins were transferred to PVDF membranes. Next the PVDF membrane was closed with skim milk and incubated with first and second antibodies. Finally, the ECL Kit (Beyotime Biotechnology, P0018FS) was added for imaging. The results calculated by Image J. The antibody information is shown in Supplementary Table 3.

### Immunofluorescence

Place prepared slides in six orifice, according to the required concentration vaccination cells to be full of 80 to 90%, with 4% paraformaldehyde fixed cells after PBS washing, 0.5% of Triton X−100 (Solarbio, T8200) room temperature appear a 10 min, 5% BSA (Solarbio, T8020) closed 30 min, incubation primary antibody (rabbit anti-SOX4) 4°C overnight, the secondary antibody was incubated after washing with PBS, DAPI (Solarbio, C0060) added and incubated in dark for 15 min, and the tablets were sealed with the blocking solution containing anti-fluorescent quench agent, and the images were collected.

### Animal Experiments

Four-week-old female Balb/c-nude mice were obtained from Slaccas (Slaccas Laboratory Animal, Shanghai, China). According to the National and Institutional Guidelines for Animal Care and Use. All experiment procedures were performed under protocols approved by the Northeast Agricultural University Ethics Committee for the use of laboratory animals. ~5 × 10^7^ CHMm^TAM^/si-lnc42060 CHMm^TAM^ cells were subcutaneously injected into each mouse to form xenografts (*n* = 8). The size of the tumor formed at the injection site, whether the animal died, whether the animal tumor ruptured and the mental state of the experimental animals were monitored every 3 days. The subcutaneous tumor of mice was measured with a vernier caliper, and then the tumor tissue of the second generation was successfully planted *in situ* in nude mice. Finally, the tumor tissue of the second generation was successfully planted *in situ* in nude mice.

### Statistical Analysis

The data is presented as the mean ± SD. Statistical comparisons between treated and control groups were calculated by Student's *t*-tests using GraphPad Prism 5, and multiple groups were determined by two-way ANOVA test. A value of *P* < 0.05 (Bar with ^*^) was considered to be significant.

## Results

### LncRNAs and miRNAs Are Associated With TAMR in CMGTs

**Figure 1:**
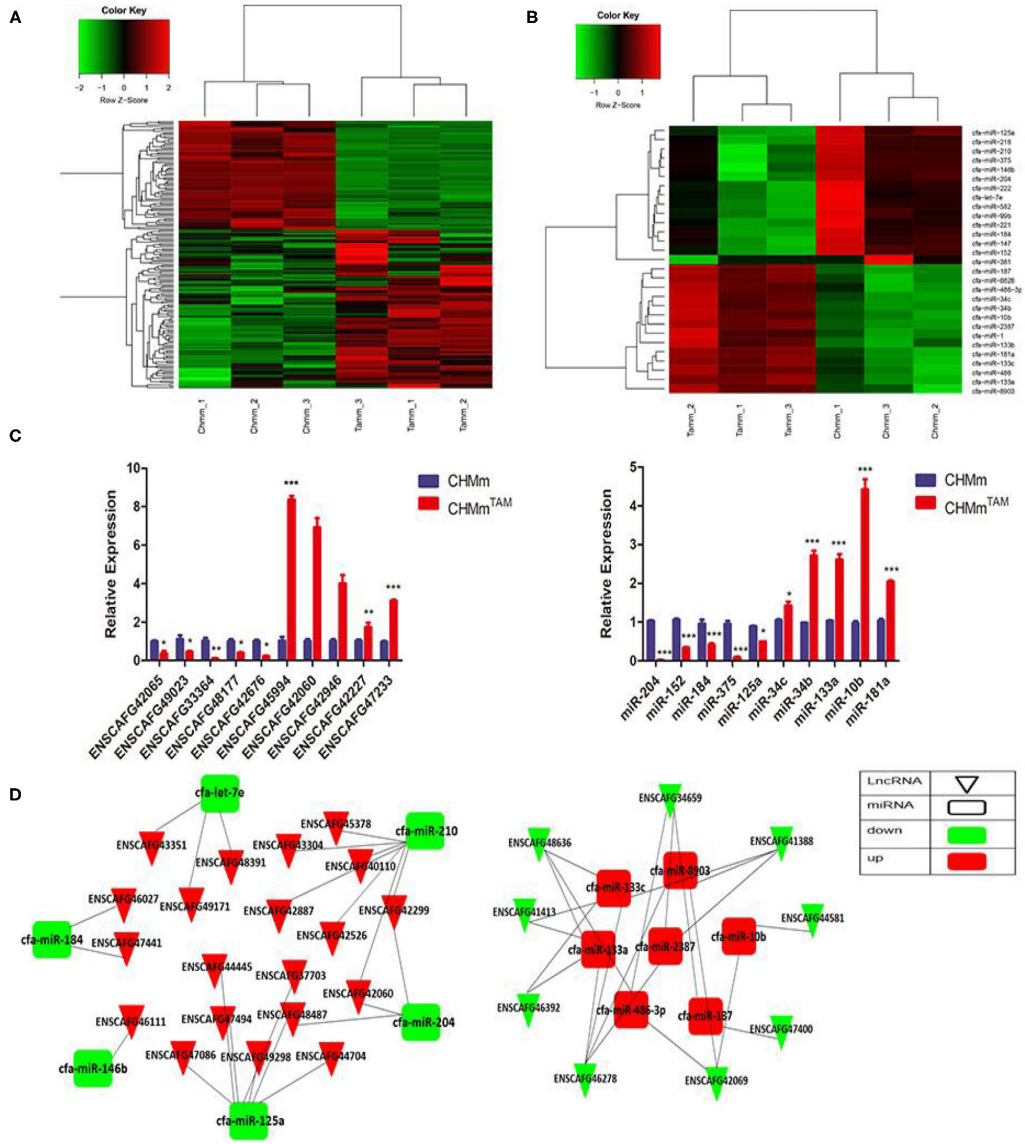
LncRNAs and miRNAs are associated with TAMR in CMGTs. **(A,B)** RNA sequencing was used to detect differentially expressed lncRNAs and miRNAs in CHMm^TAM^ and CHMm. **(C)** Expression of lncRNAs and miRNAs in CHMm^TAM^ and CHMm by qRT-PCR. **(D)** lncRNA-miRNA interaction diagram. Data are presented as means ± SD of at least three independent experiments. Compared with control group, *P < 0.05, **P < 0.01, ***P < 0.001.

To clarify the potential roles of lncRNAs and miRNAs in TamR and parental CMGTs, we analyzed the expression profiles of lncRNAs and miRNAs using transcriptome detection technology. The results showed that 162 lncRNAs and 29 miRNAs were differentially expressed (log2 fold change >1 or <-1, FDR <0.05) between TamR and parental CMGTs (Supplementary Tables 4, 5), of which 96 lncRNAs and 14 miRNAs were upregulated, and 66 lncRNAs and 15 miRNAs were downregulated in TAMRs (Figures 1A,B). The data presentedin the study are deposited in the NCBI,s Gene Expression Omnibus repository, accession number GSE164721. Subsequently, five randomly upregulated miRNAs and lncRNAs as well as 5 downregulated miRNAs and lncRNAs were verified by RT-PCR. The results were similar to the sequencing results, which confirmed the reliability of the transcriptome sequencing (Figure 1C). In this study, we constructed a hypothetical lncRNA-miRNA crosstalk network for differentially expressed miRNAs and lncRNAs, according to Miranda (Score >150, Energy <-20) and RNAhybrid (Energy <-25), and the intersection of the two algorithms was taken as the final result. Figure 1D shows the possible binding relationship between differentially expressed lncRNAs and miRNAs.

### Silencing lnc-42060 Inhibited Cell Proliferation, Migration and Restored TAM Sensitivity in TAMRs

**Figure 2:**
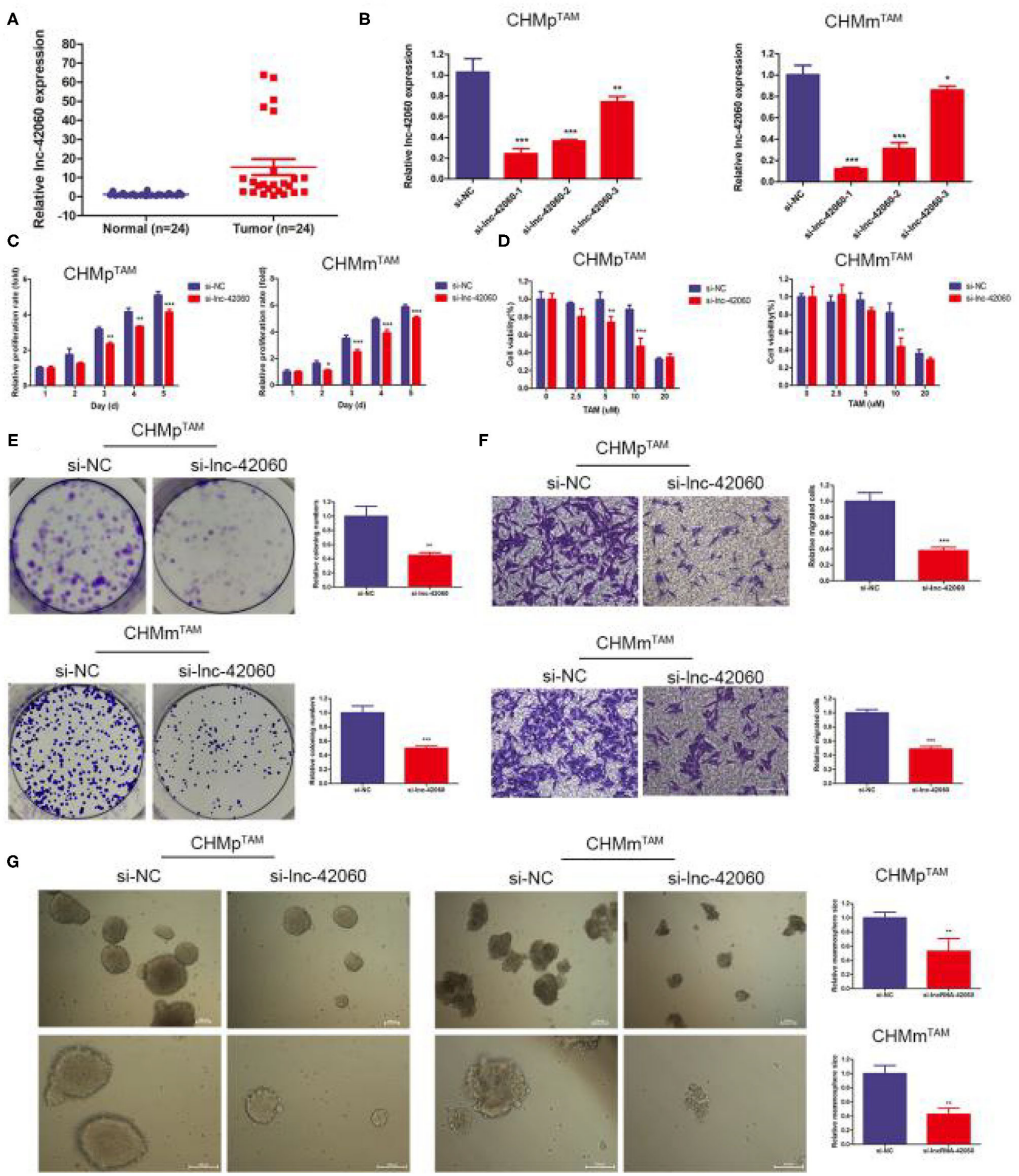
Silencing lnc-42060 inhibited cell proliferation, migration and restored TAM sensitivity in TAMRs. **(A)** Expression of lnc-42060 in CMGT and normal tissues by qRT-PCR. **(B)** The expression of lnc-42060 and GAPDH in TAMRs was determined with qRT-PCR transfected with the negative control of siRNA (si-NC) and si-lnc-42060-1/2/3. **(C)** CCK8 assay was conducted to evaluate cell proliferative ability in TAMRs/si-NC and TAMRs/si-lnc-42060. **(D)** CCK8 assay shows the variation in TAM sensitivity in TAMRs/si-NC and TAMRs/si-lnc-42060. **(E)** Colony formation is shown in TAMRs /siNC and TAMRs/si-lnc-42060. **(F)** Transwell migration was conducted to evaluate cell migration ability (magnification, 200×) in TAMRs/si-NC and TAMRs/si-lnc-42060. **(G)** Microemulsion size in TAMRs/si-NC and TAMRs/si-lnc-42060 (magnification, 100 and 200×). Data are presented as means ± SD of at least three independent experiments. Compared with control group, *P < 0.05, **P < 0.01, ***P < 0.001.

Drug-resistant cells exhibit greater proliferation, migration, and resistance to drugs. Therefore, phenotypic changes are important factors in the development of drug resistance. First, we detected lnc-42060 expression in tissues by qRT-PCR, and the results showed that lnc-42060 was highly expressed in tumor tissues (Figure 2A), which was consistent with our cell validation results. The above indicates that lnc-42060 is closely associated with tumorigenesis and drug resistance. Next, we silenced the expression of lnc-42060 in TAMRs. We designed three siRNAs, and qRT-PCR validated the knockdown efficiency. As si-lnc-42060-1 exhibited a more pronounced silencing effect, it was selected for subsequent experiments (Figure 2B). By CCK8, Transwell, and Cell cloning assay, we found that silencing lnc-42060 resulted in the inhibition of cell proliferation, migration, cloning ability, and decreased resistance to TAM (Figures 2C–F). CSCs are critical for the development of drug resistance. Sphere formation experiments showed that after silencing lnc-42060, the number of spheres formed was not significantly changed, but they became smaller (Figure 2G). In conclusion, silencing lnc-42060 can affect the biological activity of TAMRs and reverse the resistance to TAM.

### Overexpression of lnc-42060 Promoted Cell Proliferation, Migration and Increased TAMR in CMGTs

**Figure 3:**
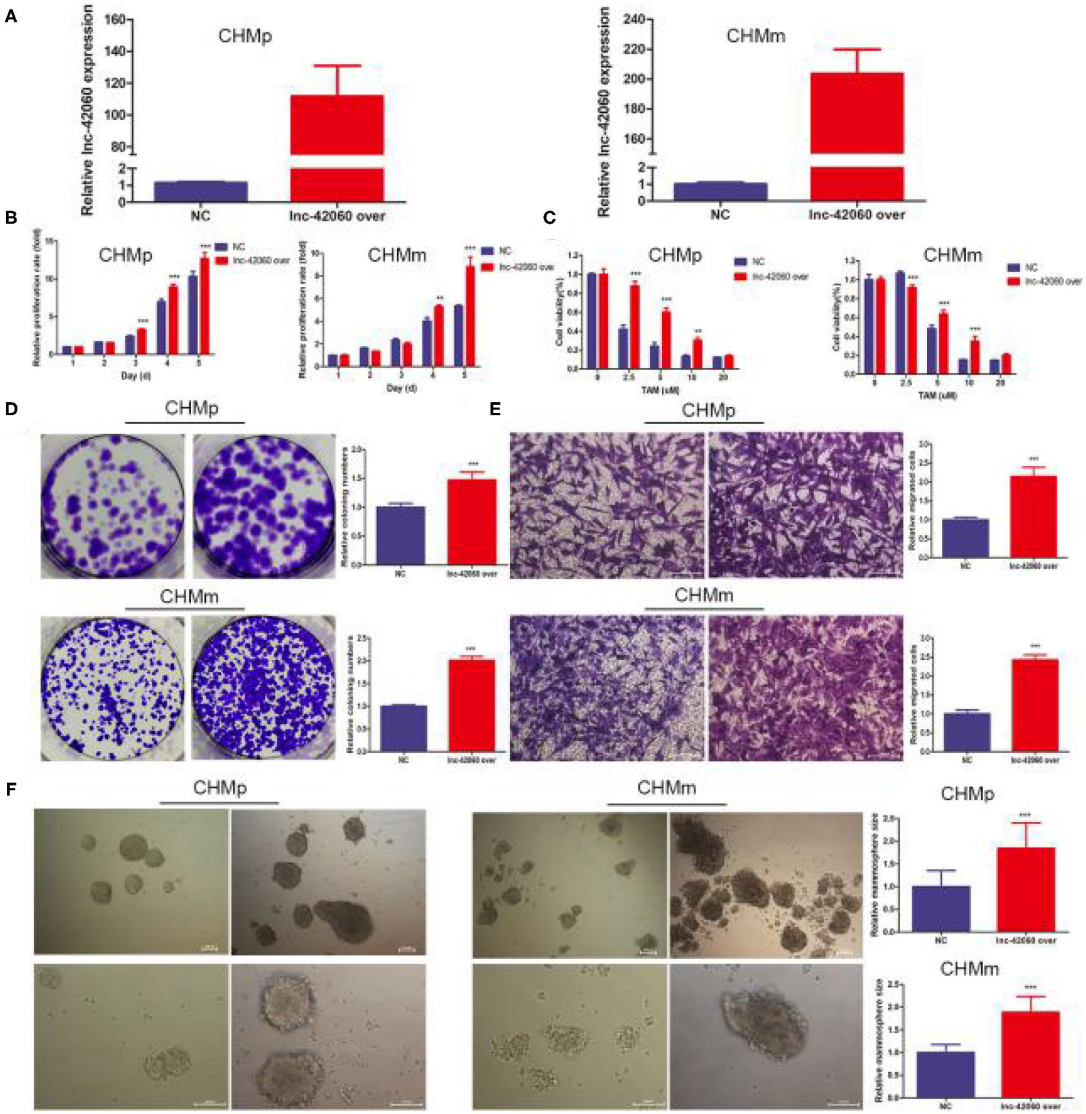
Overexpression of lnc-42060 promoted cell proliferation, migration and increased TAMR in CMGTs. **(A)** Expression of lnc-42060 in CMGTs/NC and CMGTs/lnc-42060 over. **(B)** CCK8 assay was conducted to evaluate cell proliferative ability in CMGTs/NC and CMGTs/over-lnc-42060. **(C)** Colony formation is shown in CMGTs/NC and CMGTs/lnc-42060 over. **(D)** Transwell migration was conducted to evaluate cell migration ability (magnification, 200×). **(E)** CCK8 assay shows the variation in TAM sensitivity in CMGTs/NC and CMGTs/lnc-42060 over. **(F)** Microemulsion size in CMGTs/NC and CMGTs/lnc-42060 over (magnification, 100× and 200×). Data are presented as means ± SD of at least three independent experiments. Compared with control group, **P < 0.01, ***P < 0.001.

To further validate the role of lnc-42060 in TAM drug resistance. We constructed lnc-42060-containing expression vectors to investigate the potential function and role (Figure 3A). Subsequent functional assays showed that overexpression of lnc-42060 inhibited cell viability, colony-forming and migration capacity. Simultaneous cytotoxicity assays showed that overexpression of lnc-42060 increased the resistance of CMGTs to TAM (Figures 3B–E). Based on the above experiments, we demonstrated that lnc-42060 plays a significant role in TamR and may be involved in cell proliferation, migration, clone formation and other life activities. Importantly, it can also influence the formation of stem cells (Figure 3F). Thus, lnc-42060 could serve as a tumor suppressor and an important regulator of TAM sensitivity in CMGTs.

### lncRNA-42060 Acts as an Efficient miRNA Sponge for miR-204-5p

**Figure 4:**
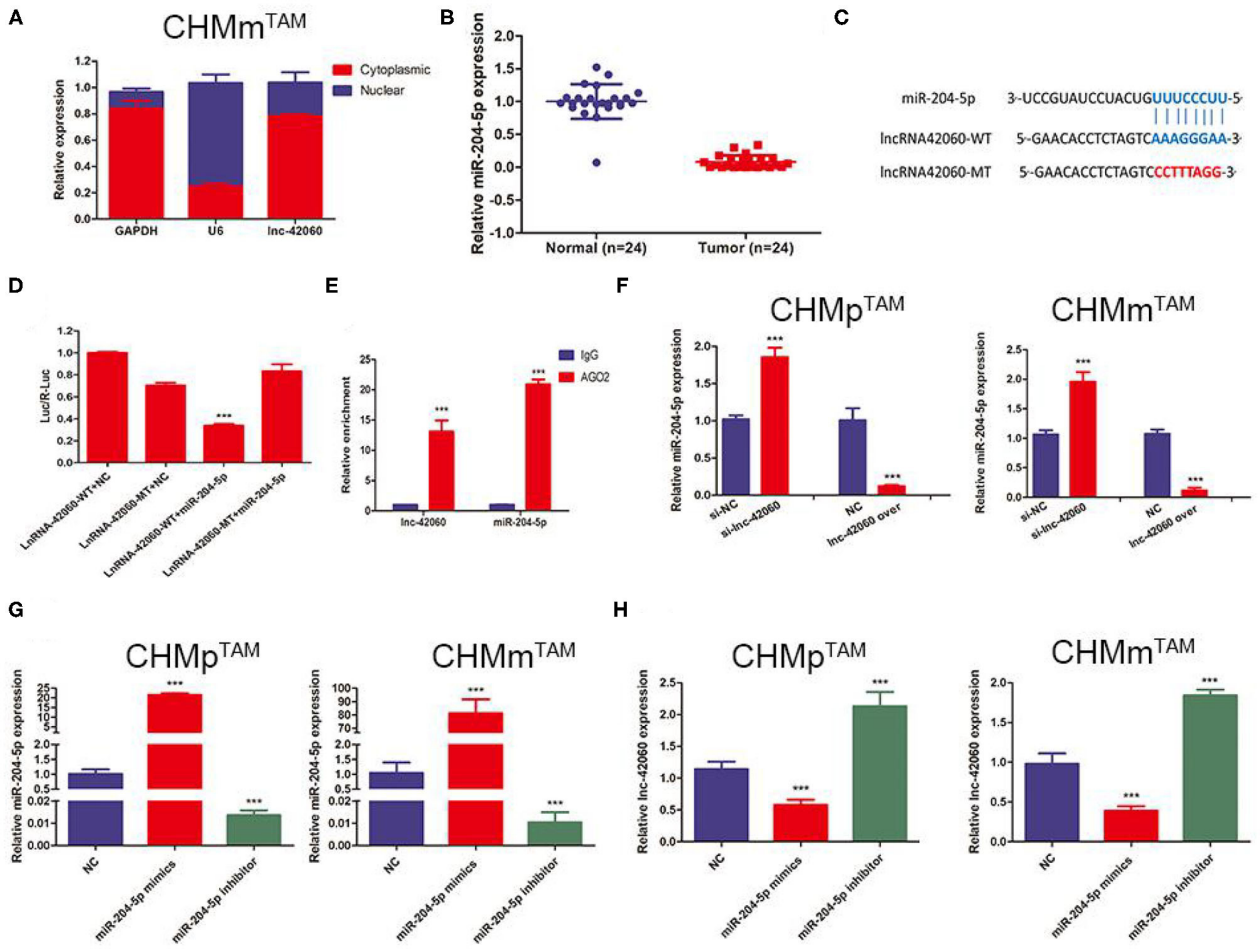
lnRNA-42060 acts an efficient miRNA sponge for miR-204-5p. **(A)** Expression of lnc-42060 in the nucleus and cytoplasm by qRT-PCR. **(B)** Expression of miR-204-5p in CMGT and normal tissues by qRT-PCR. **(C)** The predicted potential binding sites of miR-204-5p to lnc-42060 and schematic of WT and MUT of lnc-42060. **(D)** Dual-luciferase reporter assay revealed that miR-204-5p reversely controlled the luciferase activity of lnc-42060-WT, rather than MT. **(E)** The lnc-42060 and miR-204-5p levels isolated from Ago2 and IgG immunoprecipitates derived from CHMm^TAM^ cells were examined by quantitative real-time PCR. **(F–H)** Expression of lnc-42060 or miR-204-5p by qRT-PCR. Data are presented as means ± SD of at least three independent experiments. Compared with control group, ***P < 0.001.

Sequencing results detected a large number of altered lncRNAs and miRNAs, predicting a complex network of relationships between the two. lncRNAs mainly function as ceRNAs in the cytoplasm to regulate miRNA molecules, thereby influencing a range of life processes. Based on the sequencing predicted lncRNA-miRNA regulatory network, we selected lnc-42060-miR-204-5p and further verified the accuracy of sequencing and the biological processes affected. First, we detected miR-204-5p expression in tumor tissues by qRT-PCR, and we found that miR-204-5p was downregulated in tumor tissues compared to normal tissues (Figure 4A). Next, we also found that the high expression of lnc-42060 was mainly concentrated in the cytoplasm and that there were complementary binding sites with miRNA-204 (Figures 4B,C). This was also confirmed by dual-luciferase enzyme experiments. We constructed the wild-type (WT) or mutant-type (MT) of lnc-42060 with or without the binding site and incubated them with miR-204-5p, respectively. In 293T cells, a significant decrease in the activity of the luciferase reporter gene was detected after co-transfection of miR-204-5p mimic and WT, whereas the mutant vector was not significantly reduced (Figure 4D). After RNA immunoprecipitation, it was found that the amount of lnc-42060 and miR-204-5p precipitated by AGO2 antibody was significantly higher than that of IgG control group (Figure 4E), suggesting that both lnc-42060 and miR-96-5p could bind to AGO2 protein, suggesting that lnc-42060 acted on miR-204-5p through AGO2. Finally, qRT-PCR assays showed a negative correlation between miR-204-5p expression and lnc-42060 (Figures 4F–H). Taken together, these experiments suggest that lnc-42060 may act as a sponge for miR-204-5p, which is consistent with our sequencing results.

### lnc-42060 Sponges miR-204-5p to Regulate Cell Proliferation, Migration and Increased TAMR in TAMRs and CMGTs

**Figure 5:**
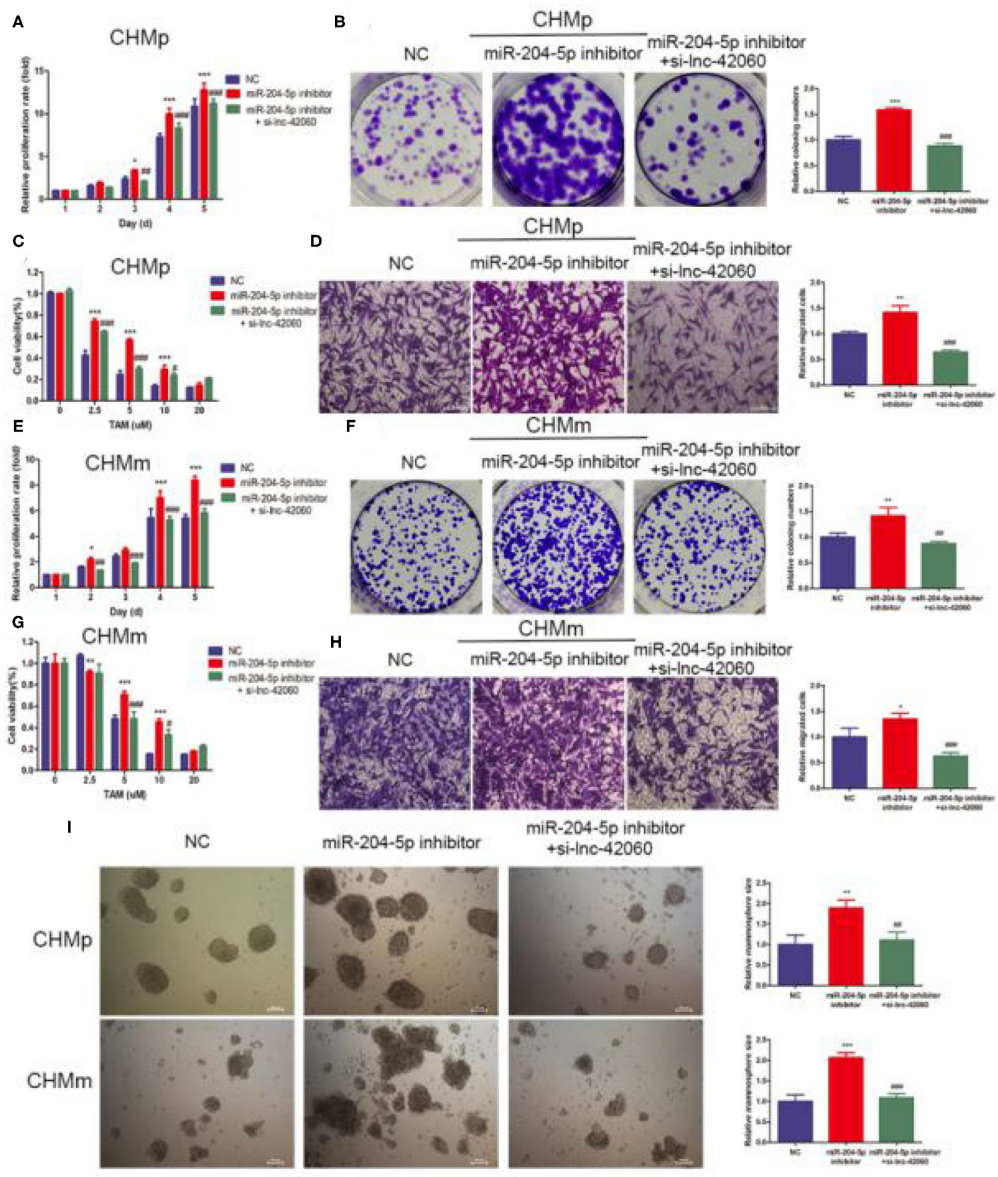
lnc-42060 sponges miR-204-5p to regulate cell proliferation, migration and increased TAMR in CMGTs. **(A,E)** CCK8 assay was conducted to evaluate cell proliferative ability in CMGTs. **(B,F)** Colony formation is shown in CMGTs. **(C,G)** CCK8 assay shows the variation in TAM sensitivity in CMGTs. **(D,H)** Transwell migration was conducted to evaluate cell migration ability (magnification, 200×) in CMGTs. **(I)** Microemulsion size in CMGTs (magnification, 200×). Data are presented as means ± SD of at least three independent experiments. Compared with control groups, *P < 0.05, **P < 0.01, ***P < 0.001; compared with miR-204 groups, ^#^*P* < 0.05, ^##^*P* < 0.01, ^###^*P* < 0.001.

**Figure 6:**
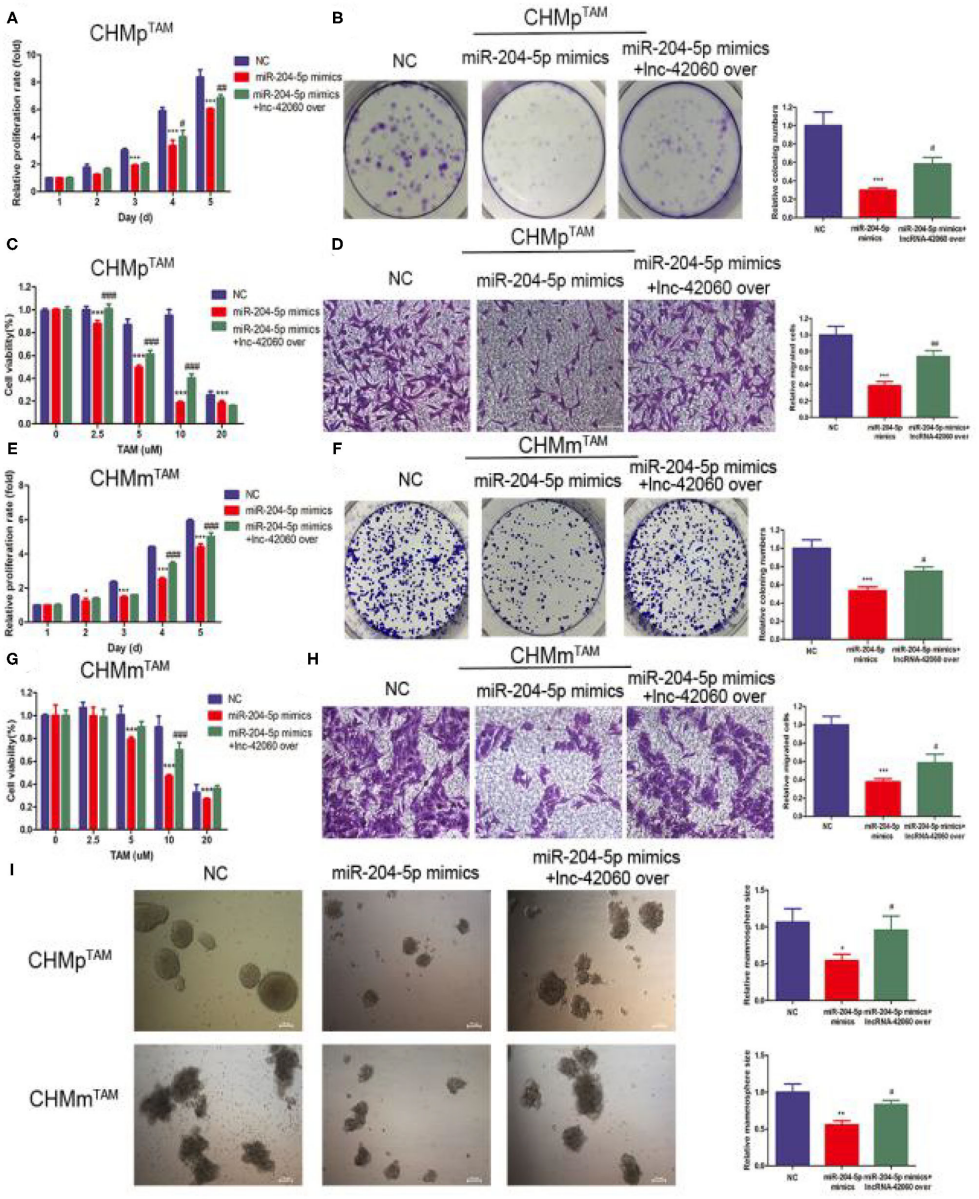
lnc-42060 sponges miR-204-5p to regulate cell proliferation, migration and increased TAMR in TAMRs. **(A,E)** CCK8 assay was conducted to evaluate cell proliferative ability in TAMRs. **(B,F)** Colony formation is shown in TAMRs. **(C,G)** CCK8 assay shows the variation in TAM sensitivity in TAMRs. **(D,H)** Transwell migration was conducted to evaluate cell migration ability (magnification, 200×) in TAMRs. **(I)** Microemulsion size in TAMRs (magnification, 200×). Data are presented as means ± SD of at least three independent experiments. Compared with control group, *P < 0.05, **P < 0.01, ***P < 0.001; compared with miR-204 group, ^#^*P* < 0.05, ^##^*P* < 0.01, ^###^*P* < 0.001.

Next, we verified the biological function of miR-204-5p. In CMGTs, inhibition of miR-204-5p activity, cell proliferation, migration, clone formation capacity, and TAMR were enhanced. This effect was attenuated if si-lnc-42060 was also silenced (Figures 5A–I). Similarly, after the addition of miR-204-5p, cell proliferation, migration, clone-forming ability, and TAMR were reduced. However, overexpression of lnc-42060 in the cells partially reversed the effect of miR-204-5p on the cells (Figures 6A–I). Taken together, these results suggest that lnc-42060 inhibits CMGT progression via miR-204-5p and sensitizes cells to TAM, thereby attenuating its oncogenic effects.

### SOX4 Is a Direct Target Gene of miR-204-5p and Is Regulated by lnc-42060

**Figure 7:**
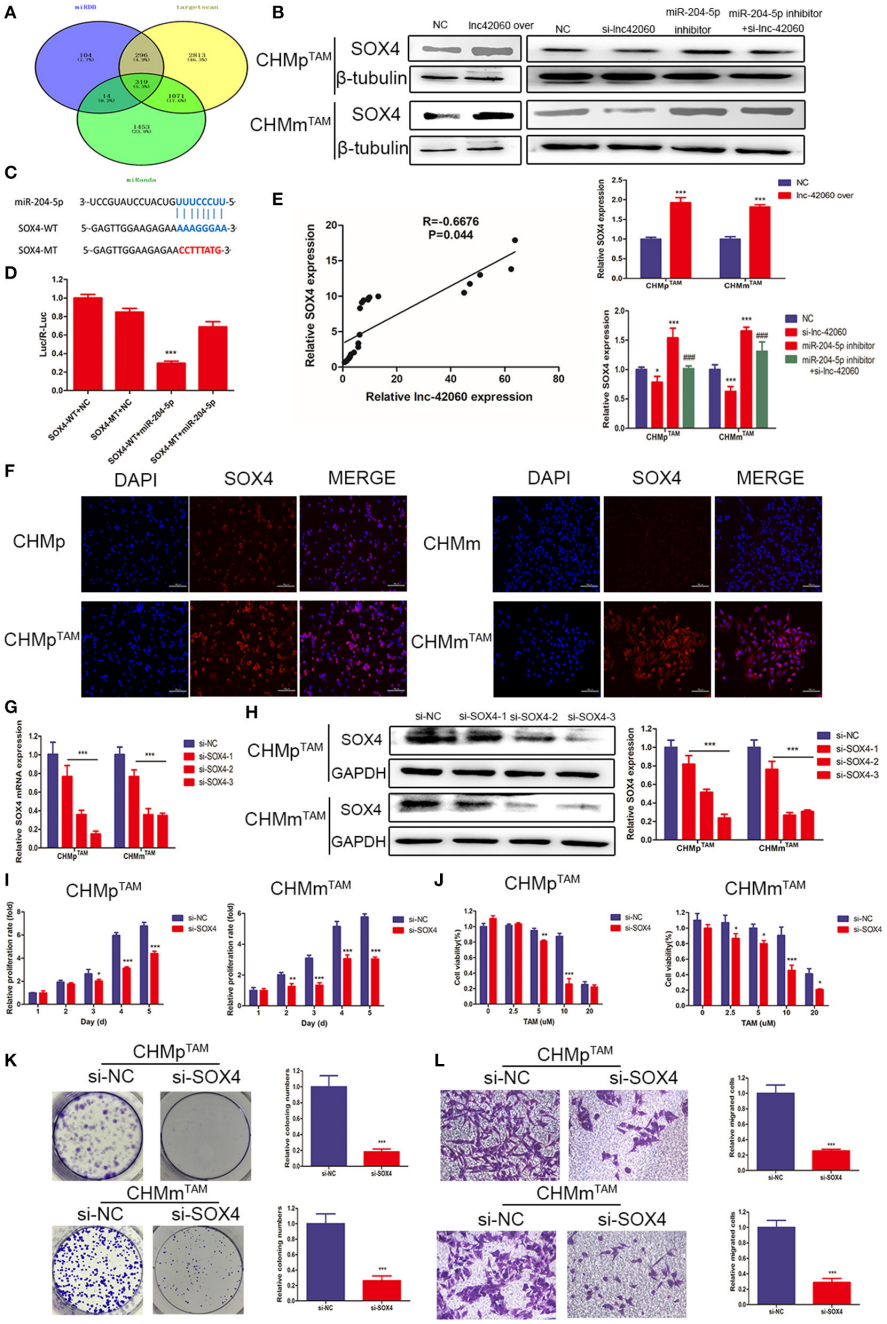
SOX4 is a direct target gene of miR-204-5P and is regulated by lnc-42060. **(A)** Venn diagram represents the mutual candidate target genes of miR-204-5p identified by miRDB, TargetScan, and miRanda. **(B)** Expression of SOX4 was tested by Western blot in TAMRs. **(C)** The predicted potential binding sites of miR-204-5p to SOX4 and schematic of WT and MT of SOX4. **(D)** Dual-luciferase reporter assay revealed that miR-204-5p reversely controlled the luciferase activity of SOX4-WT, rather than MT. **(E)** QRT-PCR was used to detect the relative expressions of lnc-42060 and SOX4. **(F)** Cellular immunofluorescence was used to detect SOX4 expression in TAMRs and CMGTs. **(G)** The expression of SOX4 in TAMRs was determined with qRT-PCR transfected with si-NC and si-lnc-42060-1/2/3. **(H)** The expression of SOX4 in TAMRs was determined with Westerin blot transfected with the negative control of si-NC and si-lnc-42060-1/2/3. **(I)** CCK8 assay was conducted to evaluate cell proliferative ability in TAMRs/si-NC and TAMRs/si-SOX4. **(J)** CCK8 assay shows the variation in TAM sensitivity in TAMRs/si-NC and TAMRs/si-SOX4. **(K)** Colony formation is shown in TAMRs/si-NC and TAMRs/si-SOX4. **(L)** Transwell migration was conducted to evaluate cell migration ability (magnification, 200×) in TAMRs/si-NC and TAMRs/si-SOX4. Data are presented as means ± SD of at least three independent experiments. Compared with control group, *P < 0.05, **P < 0.01, ***P < 0.001; compared with miR-204 group, ^###^*P* < 0.001.

We predicted potential targets (Figure 7A) for miR-204-5p using three target gene prediction databases (miRDB, TargetScan, and miRanda). The Wayne plot results showed 319 genes intersected by three algorithms. Among the next genes, SOX4 is known to be highly expressed in a variety of cancers. Therefore, we selected SOX4 as a target gene for our study. By western blotting, we found that overexpression of lnc-42060 caused an increase in SOX4 expression and that lnc-42060 silencing decreased SOX4 expression (Figure 7B), both showing the same trend. On the other hand, the addition of miR-204-5p inhibitor increased SOX4 expression, while simultaneous silencing of lnc-42060 reversed the effect of miR-204-5p inhibitor. The predicted results demonstrate the binding site of miR-204-5p to SOX4 at the 3′ UTR (Figure 7C). Dual-luciferase results showed lower luciferase activity in WT, while MT had no significant effect, indicating that SOX4 can bind to miR-204-5p and that SOX4 is an effective miR-204-5p target (Figure 7D). Finally, a positive correlation between lnc-42060 and SOX4 was detected (Figure 7E).

SOX4 can regulate multiple pathways during growth and development. Next, we tested the role of SOX4 in the tumor drug resistance process. First, we detected SOX4 expression in TAMRs and parental cells by cellular immunofluorescence, and the results showed that SOX4 fluorescence was more intense and higher in TAMRs (Figure 7F). Next, we silenced SOX4 expression in TAMRs and since si-SOX4-3 had the most significant effect, it was used in the following experiments (Figures 7G,H). Then we detected tumor progression by CCK8, Transwell, and cell cloning assays (Figures 7H–L). Taken together, these results suggest that lnc-42060, as a sponge of miR-204-5p, regulates SOX4 expression and activity, thereby further inhibiting tumor progression and sensitizing cells to TAM.

### Knocking Down lnc-42060 Reduces Tumorigenesis of CHMm^TAM^ *in vivo*

**Figure 8:**
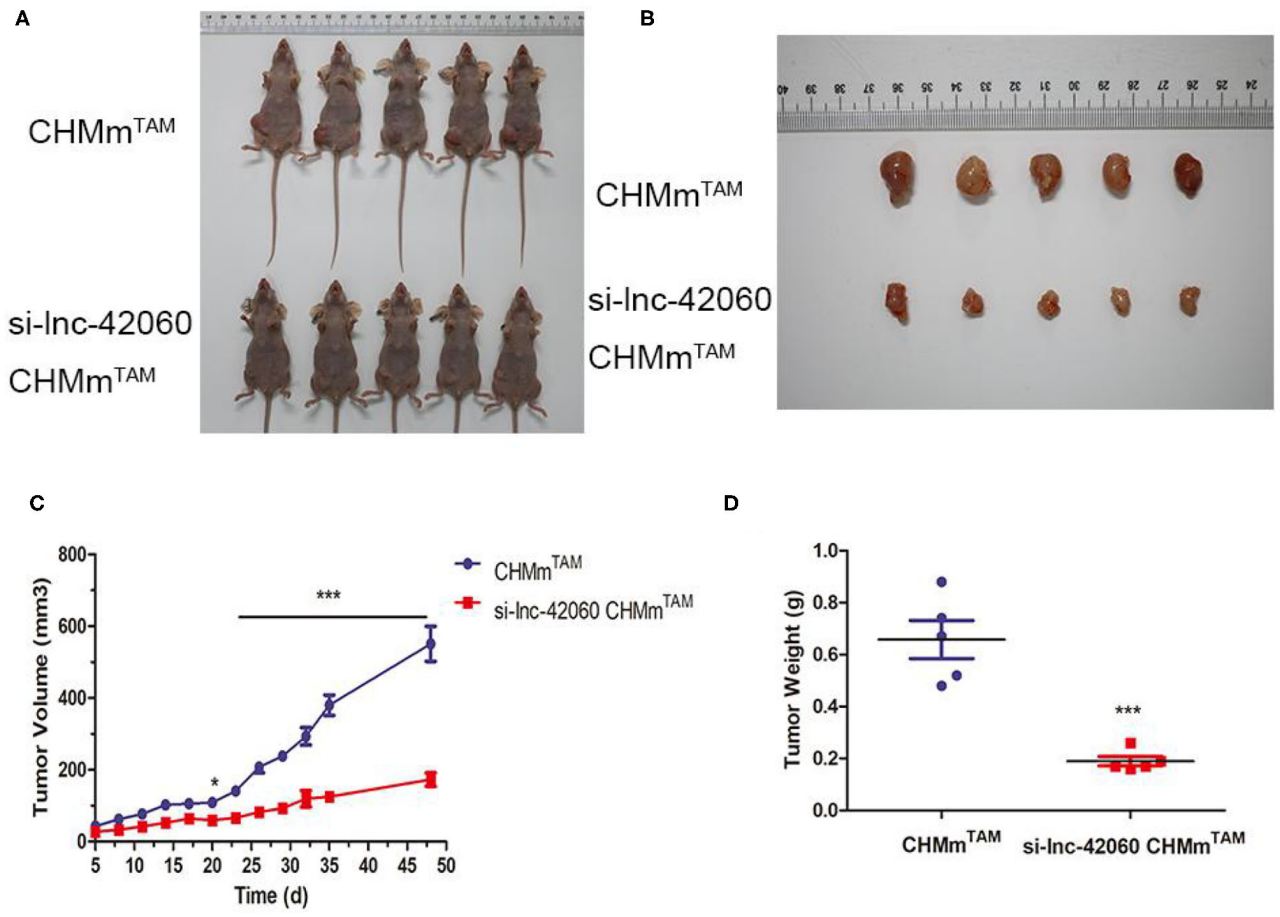
Knocking down lnc-42060 reduces tumorigenesis of CHMm^TAM^ *in vivo*. CHMm^TAM^/si-lnc-42060 CHMm^TAM^ cells infected were subcutaneously injected into nude mice. **(A,B)** Comparison of tumor size between CHMm^TAM^ and si-LNC-42060 CHMm^TAM^ cells after inoculation in mice. **(C)** Tumor volumes were examined every 3 days for 48 days. **(D)** Tumor weight were examined on day 48. Compared with control group, ***P < 0.001.

Finally, we confirmed the oncogenic effect of lnc-42060 in CMGT *in vivo*. In previous experiments, we observed that knocking down lnc-42060 could reduce the formation of cell microemulsion spheres and reduce cell stemness.To evaluate the tumor-forming ability of lnc-42060 *in vivo*, we injected different cells (CHMm^TAM^/si-42060 CHMmTAM) through fat pads, and observed and recorded the mental state, diet, and tumor size of the mice every 3 days (Figures 8A–D). The results showed that knockdown of lnc-42060 in CHMm^TAM^ cells significantly reduced the incidence of tumors, as well as tumor weight and volume, suggesting that knockdown of lnc-42060 can regulate oncogenesis *in vivo*.

## Discussion

More than 80% of breast cancer patients show ER positivity. Anti-hormonal therapy has been shown to be effective (18). Although many drugs have been developed to lower estrogen levels or block estrogen signaling, TAM remains the first choice. The emergence of resistance has reduced the relapse rate by 50% and the mortality rate by 28%, greatly limiting its clinical use (19). Therefore, it is important to determine the mechanism of TAMR and to screen new biomarkers to predict the effect of TAM treatment and improve patient survival.

LncRNAs have a non-negligible role in the diagnosis and treatment of BC. Resistance to endocrine therapy is one of the major therapeutic problems in BC. Therefore, lncRNAs modulate endocrine therapy resistance in BC and may be a key factor in the treatment of endocrine therapy resistance. lncRNA BCAR4, identified in the ZR-75-1 BC cell screen, acts as an ERBB2/ERBB3 signaling driver oncogene and promotes estrogen-independent growth and anti-estrogen resistance in BC cells (20). Another study showed that lncRNA HOTAIR may confer TAMR through ER signaling (21). Although we have elucidated the role of some lncRNAs in endocrine resistance in BC, lncRNA expression is often highly cell-type-and tissue-specific. Therefore, it is important to screen more lncRNAs from multiple perspectives and investigate their functions in the development of cancer. In this study, we performed the first CMGT endocrine TAMR-associated lncRNA expression profiling of constructed CHMm^TAM^ and parental CHMm cell lines and found 162 differentially expressed lncRNAs. We focused on the novel lnc-42060 located on chromosome 25 (25:9055794-9074347), with a length of 1577bp. It is upregulated in mammary tissues. Furthermore, our results indicate that silencing or overexpression of lnc-42060 inhibits the proliferation, migration, and cloning of TAMRs or parental cells. This was accompanied by changes in EMT and CSC characteristics. These results indicate that lncRNAs play a non-negligible role in drug resistance and, importantly, the novel ENSCAFG42060 was found to play an oncogenic role.

There are 27,919 lncRNAs that have been identified through many databases of transcriptome data and cluster gene expression analyses, 69% of which are implied with potential function (22). The diverse functions of lncRNAs are influenced by different subcellular localizations. In the nucleus, lncRNAs can not only regulate epigenetic inheritance by interactions with chromatin remodeling complexes but also regulate transcription through binding to general transcriptional mechanisms or specific regulators. In the cytoplasm, lncRNAs usually function as ceRNAs, influencing the status of mRNAs and making a lncRNA-miRNA-mRNA close linkage, which is also the most extensively studied mode of action. CeRNAs compete for shared miRNAs. They regulate each other at the post-transcriptional level, linking the functions of protein-coding mRNAs to non-coding RNA (23, 24). RNA sponges are commonly used to describe this phenomenon. For example, Li et al. found that the new lnc-000052 could pass through the miR-96-5P-PIK3R1 molecular axis resulting in reduced stem cell formation in osteoporotic rats. Lu et al. (25) showed that in BC, the lncRNA LINC00511 was found to affect E2F1 by interacting with miR-185, which promotes cancer cell proliferation, invasion, and migration. Yao et al. (26) also demonstrated that NONHSAT10109 can target Twist1 by sponging miR-129-5p to play the role of ceRNA in BC cells. Based on the subcellular location of lnc-42060 in TAMRs (mainly in the cytoplasm), we propose a possible mechanism of its post-transcriptional regulation, including the ceRNA network.

The lncRNA HULC is highly upregulated in hepatocellular carcinoma and has multiple binding sites with miR-372, which results in reduced translation of the target gene PRKACB (27). In another study, Tang and colleagues (27) detected changes in lncRNA expression in BC tissues and stem cells and found that down-regulation of lncRNA CCAT1 was associated with multiple miRNA molecules such as miR-204 and miR-148. This suggests that ce-RNA networks (ce-RNETs) are complex and variable, and we should expand our study sample, which is helpful in discovering more target factors. Therefore, we also tested miRNA expression profiles and interactions with lncRNAs (Figures 1A-D). The results showed that there were 29 differentially expressed miRNAs in CHMm^TAM^ and CHMm. In addition, lncRNA-miRNA interactions also showed complex regulatory relationships. Among them, miR-204 was the most significantly downregulated. Many studies have shown that low expression of miR-204-5p is critical for the control of cell growth, cell apoptosis, epithelial-mesenchymal transition, angiogenesis, and sensitivity to chemotherapy. Hong et al. (28) demonstrated that miRNA-204-5p acts as a tumor suppressor to regulate BC growth, metastasis, and immune microenvironment reconstruction. Wa and colleagues (29) have demonstrated that miR-204-5p promotes bone metastasis via the NF-κB signaling pathway. However, in other studies, miR-204 has shown proliferation promotion effects in cancer cells, indicating that miR-204-5p may play double regulatory roles (30). Therefore, it is necessary to test the expression of miR-204-5p in a variety of breast tumor cells to explore its regulatory mechanism. Currently, miR-204-5p has never been explored in CMGT. In our study, miR-204-5p, corresponding to lnc-42060, was further analyzed. Luciferase reporter gene experiments and RNA immunoprecipitation further confirmed that miR-204-5p is a potential target ceRNA of lnc-42060 in TAMRs. Lnc-42060 and miR-204-5p clearly inhibit each other in TAMRs. It is important that silencing or overexpression of miR-204-5p partially reversed the drug resistance, migration, and cloning of TAM mediated by ENSCAFG42060 knockdown or overexpression. From these observations, we conclude that lnc-42060 can function as a ceRNA in CMGTs by sponging miR-204-5p.

SOX4 is a key transcription factor in many developmental processes and is overexpressed in more than 20 types of malignancies. Many studies have confirmed that SOX4 is an oncogene. Its overexpression is associated with abnormal gene amplification and activation of the PI3K, Wnt, and TGF pathways (31). The overexpression of SOX4 can promote cell survival, CSC, EMT, migration and metastasis, and drug resistance, suggesting that SOX4 is associated with a poor prognosis for disease. Importantly, some scholars have also determined that SOX4 participates in ceRNA regulation. Zhao et al. found that miR-140-5p decreased when SOX4 increased in melanoma tissues and cells, which indicated that there is a direct interaction between them (32). In addition, Lu et al. (33) demonstrated that the lncRNA DSCAM-AS1 promotes proliferation and migration of colon cancer cells by regulating the miR-204-SOX4 axis. However, only the effect of SOX4 on cell migration was discussed in this study. With the progress of medical treatment, more and more attention has been paid to the occurrence and development of canine diseases, not only because they can serve as human life partners, but also because they are very similar to humans in terms of gene structure and physiological environment, making them a good animal model (34–36).

At present, the involvement of SOX4 in ceRNA in BC remains to be confirmed, and it was reported for the first time in CMGT. In this study, we further identified SOX4 as a target protein of the ENSCAFG42060/miR-204-5p axis based on the following evidence. First, bioinformatics prediction, qRT-PCR, western blot, and dual-luciferase reporter assay indicated that SOX4 is a direct target gene of miR-204-5p in TAMRs. Second, the expression of lnc-42060 and SOX4 in CMGT tissue was significantly positively correlated. Lnc-42060 positively regulates the expression of SOX4 at both the mRNA and protein levels in TAMRs. Third, SOX4 silencing significantly inhibited the proliferation, migration, and clonal formation of TAMRs. In conclusion, this study focuses on novel lncRNAs involved in TAMR. For the first time, we identified lnc-42060/miR-204-5p by screening the expression profiles and the interaction relationship between lncRNA and miRNA in parental cell lines and TAMRs. Subsequently, we found that lnc-42060 was upregulated in CMGT tissues and TAMRs, and inter-restricted with miR-204 to alter cell proliferation, migration ability, and sensitivity to TAM. Further cell biology experiments revealed that lnc-42060 promoted drug resistance, proliferation, and metastasis of TAMRs through the miR-204-5p/SOX4 axis. That is, lnc-42060 and miR-204-5p are regarded as ceRNAs that regulate the expression of SOX4. Therefore, our findings illustrate that the novel lnc-42060 is a tumor promoter and potential BC therapeutic biomarker.

## Data Availability Statement

The datasets presented in this study can be found in online repositories. The names of the repository/repositories and accession number(s) can be found at: NCBI's Gene Expression Omnibus (GSE164721).

## Ethics Statement

The animal study was reviewed and approved by Northeast Agricultural University, Harbin, Heilongjiang Province. Written informed consent was obtained from the owners for the participation of their animals in this study.

## Author Contributions

EX and YL contributed conception and design of the study. MH, DT, and YF conducted experiments. EX organized the database. XR performed the statistical analysis. EX wrote the first draft of the manuscript. RG has completed part of the *in vivo* test. All authors contributed to manuscript revision, read, and approved the submitted version.

## Conflict of Interest

The authors declare that the research was conducted in the absence of any commercial or financial relationships that could be construed as a potential conflict of interest.
