# Supplementary material for: LncRNA-42060 Regulates Tamoxifen Sensitivity and Tumor Development via Regulating the miR-204-5p/SOX4 Axis in Canine Mammary Gland Tumor Cells
Source: Front Vet Sci. 2021 Jun 21;8:654694. doi: 10.3389/fvets.2021.654694 (PMC8255626; doi:10.3389/fvets.2021.654694)
Supplement: Supplementary file 1 [file Table_1.docx]

**Supplementary table 1 The primers for qPCR**

| Genes | Sequence (5′→3′) |
| --- | --- |
| lnc-42065 | Forward: GAGTGAAGACAGCAGAGGCATTG |
|  | Reverse: GCCGTCCTGTTGTGCAGAATT |
| lnc-49023 | Forward: CCAAGCAGGGACTTGTTGCC |
|  | Reverse: GGTGGAGAAGGAGCCAGGAAA |
| lnc-33364 | Forward: TTCTAAAGCCCTGCCAATACT |
|  | Reverse: GCCTCTTCCTCTTGTGCTGTCTA |
| lnc-48177 | Forward: AGGCAGTGTAATTAGCTGATTGTCA |
|  | Reverse: TGACAATCAGCTAATTACACTGCCT |
| lnc-42676 | Forward: CCTGAGACCACCTGGACTGTTT |
|  | Reverse: CCAGCCTTTATCATGGTGAATT |
| lnc-45994 | Forward: CCCCAGAGCTTCCCTACCCA |
|  | Reverse: CTCCTGCAAACCACAAATGTCC |
| lnc-42060 | Forward: TGGCTGGGCAGTAGACACGA |
|  | Reverse: CAACTGGCTCCCGATACAAACAT |
| lnc-42946 | Forward: ATGGGCGGGGCCAGGGCGGGGGCGG |
|  | Reverse: CCGCCCCCGCCCTGGCCCCGCCCAT |
| lnc-42227 | Forward: TGCCCTCCCTCTTCTGTTGC |
|  | Reverse: GGTGGGAATGGACCTGTGGT |
| lnc-47233 | Forward: AGATGCCGTGTCCTTGTATGC |
|  | Reverse: TCTTCACCTTGCTTTCCCAGTC |
| miR-204 | GCCCGTTCCCTTTGTCATCCTA |
| miR-152 | AGGCAGTGTAATTAGCTGATTGTCA |
| miR-184 | CTAAGTGTTGGACGGAGAACTGATA |
| miR-375 | GGACCTGAGCGTTTTGTTCGTTC |
| miR-125 | CTTTAACCTGTGAGGACATCCAGGG |
| miR-34c | CGGAGGCAGTGTAGTTAGCTGAT |
| miR-34b | AGGCAGTGTAATTAGCTGATTG |
| miR-133a | TGCTTTGCTAGAGCTGGTAAAATGG |
| miR-10b | CCCTGTAGAACCGAATTTGTGTG |
| miR-181a | CGGAACATTCAACGCTGTCGGT |
| SOX4 | Forward: GAAGACGACCTGCTCGACCTGA |
|  | Reverse: GCGTGCAGTAGTCCGGGAAC |
