# Supplementary material for: LncRNA-42060 Regulates Tamoxifen Sensitivity and Tumor Development via Regulating the miR-204-5p/SOX4 Axis in Canine Mammary Gland Tumor Cells
Source: Front Vet Sci. 2021 Jun 21;8:654694. doi: 10.3389/fvets.2021.654694 (PMC8255626; doi:10.3389/fvets.2021.654694)
Supplement: Supplementary file 2 [file Table_2.docx]

**Supplementary table 2 Lnc-42060 and SOX4 wild type/mutant type sequences**

| 3’UTR | Forward（5’-3’） | Reverse（5’-3’） |
| --- | --- | --- |
| SOX4-  pMIR-REPORT-WT | ACGCGTCGGGCGCGCGCGCGCGGGCCGGGGGCCGGGGGCCGCGGGCCGGGGGGCGGGGGGCGGGCGAGGAGGAGGAAGGAGGAGAAAAATTAAGAAGAGCGCGAGAGAAGTGGACACAGGACGAGTTGGAAGAGAA**AAAGGGA**ACGGGAAAAAATTAAAAAGGAAAAGGAAAAAAAAAAAAAAAGAGAGAGAGAGAAAAGTGAGCAGGGAAGCTT | AAGCTTCCCTGCTCACTTTTCTCTCTCTCTCTTTTTTTTTTTTTTTCCTTTTCCTTTTTAATTTTTTCCCG**TTCCCTTT**TTCTCTTCCAACTCGTCCTGTGTCCACTTCTCTCGCGCTCTTCTTAATTTTTCTCCTCCTTCCTCCTCCTCGCCCGCCCCCCGCCCCCCGGCCCGCGGCCCCCGGCCCCCGGCCCGCGCGCGCGCGCCCGACGCGT |
| SOX4-  pMIR-REPORT-MT | ACGCGTCGGGCGCGCGCGCGCGGGCCGGGGGCCGGGGGCCGCGGGCCGGGGGGCGGGGGGCGGGCGAGGAGGAGGAAGGAGGAGAAAAATTAAGAAGAGCGCGAGAGAAGTGGACACAGGACGAGTTGGAAGAGAA**CCTTTATG**CGGGAAAAAATTAAAAAGGAAAAGGAAAAAAAAAAAAAAAGAGAGAGAGAGAAAAGTGAGCAGGGAAGCTT | AAGCTTCCCTGCTCACTTTTCTCTCTCTCTCTTTTTTTTTTTTTTTCCTTTTCCTTTTTAATTTTTTCCCG**CATAAAGG**TTCTCTTCCAACTCGTCCTGTGTCCACTTCTCTCGCGCTCTTCTTAATTTTTCTCCTCCTTCCTCCTCCTCGCCCGCCCCCCGCCCCCCGGCCCGCGGCCCCCGGCCCCCGGCCCGCGCGCGCGCGCCCGACGCGT |
| lnc-42060-  pMIR-  REPORT-WT | ACGCGTCTTCTCCCAAAGGTGTCCTTGTATTATATCTTTTGAGCAAATGAGCGAACACCTCTAGTC**AAAGGGAA**ATGAATAGTACCAGGTGAATCCCACCCTGTGGGGAGGTTTTATTGACGTGCCAAGCTT | AAGCTTGGCACGTCAATAAAACCTCCCCACAGGGTGGGATTCACCTGGTACTATTCAT**TTCCCTTT**GACTAGAGGTGTTCGCTCATTTGCTCAAAAGATATAATACAAGGACACCTTTGGGAGAAGACGCGT |
| lnc-42060-  pMIR-  REPORT-WT | ACGCGTCTTCTCCCAAAGGTGTCCTTGTATTATATCTTTTGAGCAAATGAGCGAACACCTCTAGTC**CCTTTAGG**ATGAATAGTACCAGGTGAATCCCACCCTGTGGGGAGGTTTTATTGACGTGCCAAGCTT | AAGCTTGGCACGTCAATAAAACCTCCCCACAGGGTGGGATTCACCTGGTACTATTCATC**CTAAAGGG**ACTAGAGGTGTTCGCTCATTTGCTCAAAAGATATAATACAAGGACACCTTTGGGAGAAGACGCGT |
