# Supplementary material for: LncRNA-42060 Regulates Tamoxifen Sensitivity and Tumor Development via Regulating the miR-204-5p/SOX4 Axis in Canine Mammary Gland Tumor Cells
Source: Front Vet Sci. 2021 Jun 21;8:654694. doi: 10.3389/fvets.2021.654694 (PMC8255626; doi:10.3389/fvets.2021.654694)
Supplement: Supplementary file 3 [file Table_3.docx]

**Supplementary table 3 The antibodies used in the present study**

| Antibodies | Dilution ratio | kD | Resource |
| --- | --- | --- | --- |
| SOX4 | 1:500 | 52 | Bioss, China |
| E-cadherin | 1:500 | 135 | Wanleibio, China |
| Vimentin | 1:500 | 54 | Bioss, China |
| ZEB1 | 1:300 | 124 | Wanleibio, China |
| SOX2 | 1:1000 | 34 | ABclonal Biotechnology |
| OCT4 | 1:500 | 52 | Wanleibio, China |
| Nanong | 1:500 | 40 | Bioss, China |
| GAPDH | 1:500 | 37 | Wanleibio, China |
