# Supplementary material for: LncRNA-42060 Regulates Tamoxifen Sensitivity and Tumor Development via Regulating the miR-204-5p/SOX4 Axis in Canine Mammary Gland Tumor Cells
Source: Front Vet Sci. 2021 Jun 21;8:654694. doi: 10.3389/fvets.2021.654694 (PMC8255626; doi:10.3389/fvets.2021.654694)
Supplement: Supplementary file 4 [file Table_4.docx]

**Supplementary table 4 Differentially expressed of lncRNAs in TAMRs and CMGTs**

| AccID | log2FC | FDR | Style |
| --- | --- | --- | --- |
| ENSCAFG00000046951 | 6.699187516 | 1.19276E-06 | up |
| ENSCAFG00000044485 | 6.023068811 | 3.90261E-05 | up |
| ENSCAFG00000044352 | 5.679429035 | 0.000187704 | up |
| ENSCAFG00000037703 | 5.586191964 | 6.50164E-05 | up |
| ENSCAFG00000045994 | 5.501064465 | 9.1991E-124 | up |
| ENSCAFG00000047441 | 4.798123793 | 0.006591837 | up |
| ENSCAFG00000042060 | 4.466095055 | 0.00493413 | up |
| ENSCAFG00000038338 | 4.378705291 | 5.48936E-08 | up |
| ENSCAFG00000047454 | 4.067568245 | 0.014950011 | up |
| ENSCAFG00000040110 | 3.976625102 | 0.021613682 | up |
| ENSCAFG00000042887 | 3.936969189 | 0.021373365 | up |
| ENSCAFG00000047494 | 3.919989594 | 0.033064718 | up |
| ENSCAFG00000048222 | 3.909269469 | 0.023115499 | up |
| ENSCAFG00000049871 | 3.878170643 | 1.81712E-11 | up |
| ENSCAFG00000042475 | 3.732655573 | 0.00912994 | up |
| ENSCAFG00000045299 | 3.681642246 | 0.040650105 | up |
| ENSCAFG00000043518 | 3.656152716 | 0.001642195 | up |
| ENSCAFG00000037845 | 3.614095749 | 0.001642195 | up |
| ENSCAFG00000036451 | 3.286430013 | 5.21065E-13 | up |
| ENSCAFG00000047849 | 3.128636878 | 4.02548E-08 | up |
| ENSCAFG00000041736 | 3.072801828 | 0.001785567 | up |
| ENSCAFG00000042946 | 2.86617929 | 6.88156E-31 | up |
| ENSCAFG00000043382 | 2.84180001 | 0.002898169 | up |
| ENSCAFG00000041398 | 2.717925877 | 0.026208115 | up |
| ENSCAFG00000049003 | 2.663529841 | 0.030697685 | up |
| ENSCAFG00000044851 | 2.631424414 | 0.018594415 | up |
| ENSCAFG00000042299 | 2.56339643 | 0.008998626 | up |
| ENSCAFG00000036080 | 2.497696885 | 0.024186198 | up |
| ENSCAFG00000045272 | 2.485878828 | 0.000124209 | up |
| ENSCAFG00000036684 | 2.440584218 | 0.044959879 | up |
| ENSCAFG00000048722 | 2.366240872 | 0.035966328 | up |
| ENSCAFG00000043351 | 2.272526702 | 0.017548539 | up |
| ENSCAFG00000032673 | 2.271783835 | 2.66238E-12 | up |
| ENSCAFG00000047060 | 2.268101058 | 0.009973381 | up |
| ENSCAFG00000044250 | 2.249185402 | 0.004705549 | up |
| ENSCAFG00000043195 | 2.215584707 | 0.020540377 | up |
| ENSCAFG00000042526 | 2.178637562 | 0.008244539 | up |
| ENSCAFG00000047086 | 2.1749548 | 0.037924906 | up |
| ENSCAFG00000046000 | 2.13197074 | 0.017254207 | up |
| ENSCAFG00000045306 | 2.08175624 | 0.000429918 | up |
| ENSCAFG00000045643 | 2.022118802 | 0.000303189 | up |
| ENSCAFG00000044949 | 1.982444591 | 0.019381792 | up |
| ENSCAFG00000038442 | 1.961011448 | 7.78169E-12 | up |
| ENSCAFG00000042204 | 1.954732445 | 0.028104483 | up |
| ENSCAFG00000046287 | 1.878238652 | 2.54023E-05 | up |
| ENSCAFG00000048313 | 1.824249999 | 0.029576442 | up |
| ENSCAFG00000041906 | 1.822158547 | 0.033938716 | up |
| ENSCAFG00000045838 | 1.8006742 | 0.048384026 | up |
| ENSCAFG00000046856 | 1.797598632 | 0.0128112 | up |
| ENSCAFG00000046111 | 1.796116338 | 0.00332645 | up |
| ENSCAFG00000049171 | 1.790788469 | 0.033187013 | up |
| ENSCAFG00000042227 | 1.781315591 | 3.9719E-05 | up |
| ENSCAFG00000019497 | 1.776770882 | 1.63948E-09 | up |
| ENSCAFG00000048391 | 1.776243067 | 0.024688087 | up |
| ENSCAFG00000019827 | 1.74135291 | 4.31418E-24 | up |
| ENSCAFG00000033387 | 1.736601954 | 0.017291438 | up |
| ENSCAFG00000047526 | 1.711329596 | 0.027212679 | up |
| ENSCAFG00000023156 | 1.694547012 | 0.01746571 | up |
| ENSCAFG00000046027 | 1.644570193 | 9.69726E-08 | up |
| ENSCAFG00000046882 | 1.606695246 | 0.042273438 | up |
| ENSCAFG00000019252 | 1.603110995 | 1.11589E-18 | up |
| ENSCAFG00000049298 | 1.593169059 | 1.43334E-05 | up |
| ENSCAFG00000049729 | 1.582318947 | 0.021891015 | up |
| ENSCAFG00000045374 | 1.569547634 | 0.018202457 | up |
| ENSCAFG00000025925 | 1.551652764 | 2.76004E-99 | up |
| ENSCAFG00000047233 | 1.551403504 | 0.00434208 | up |
| ENSCAFG00000049195 | 1.54917305 | 1.6337E-13 | up |
| ENSCAFG00000041451 | 1.532947676 | 2.19352E-05 | up |
| ENSCAFG00000044704 | 1.512843971 | 2.97752E-06 | up |
| ENSCAFG00000047822 | 1.482132959 | 0.021804615 | up |
| ENSCAFG00000041061 | 1.435149155 | 1.86316E-82 | up |
| ENSCAFG00000044445 | 1.432392762 | 2.53697E-13 | up |
| ENSCAFG00000038146 | 1.405045572 | 7.85566E-06 | up |
| ENSCAFG00000043835 | 1.402311013 | 0.042105978 | up |
| ENSCAFG00000046926 | 1.398721577 | 2.23433E-17 | up |
| ENSCAFG00000049260 | 1.396899009 | 0.007003111 | up |
| ENSCAFG00000046375 | 1.367852789 | 0.02934522 | up |
| ENSCAFG00000046998 | 1.35046068 | 0.024637072 | up |
| ENSCAFG00000043304 | 1.350142949 | 0.038579025 | up |
| ENSCAFG00000043123 | 1.305752613 | 0.004653145 | up |
| ENSCAFG00000043795 | 1.266208067 | 0.001585661 | up |
| ENSCAFG00000050018 | 1.266102332 | 0.001685739 | up |
| ENSCAFG00000049022 | 1.263743771 | 1.06892E-11 | up |
| ENSCAFG00000031575 | 1.177557019 | 3.18998E-09 | up |
| ENSCAFG00000001569 | 1.177349414 | 5.73106E-36 | up |
| ENSCAFG00000048763 | 1.156461254 | 7.30326E-16 | up |
| ENSCAFG00000045378 | 1.133459413 | 0.000945132 | up |
| ENSCAFG00000044809 | 1.129812585 | 0.000564576 | up |
| ENSCAFG00000034538 | 1.107211031 | 1.20616E-05 | up |
| ENSCAFG00000025850 | 1.091266324 | 4.28465E-22 | up |
| ENSCAFG00000029302 | 1.083749888 | 1.80959E-35 | up |
| ENSCAFG00000048487 | 1.073526673 | 0.016827589 | up |
| ENSCAFG00000045499 | 1.047740557 | 0.003503892 | up |
| ENSCAFG00000050079 | 1.022684901 | 0.021829006 | up |
| ENSCAFG00000028156 | 1.006507748 | 8.79754E-30 | up |
| ENSCAFG00000037303 | 1.000119453 | 2.24328E-15 | up |
| ENSCAFG00000029101 | -1.042635979 | 0.000377771 | down |
| ENSCAFG00000044175 | -1.051596351 | 2.64836E-40 | down |
| ENSCAFG00000041177 | -1.076609891 | 2.29008E-07 | down |
| ENSCAFG00000048640 | -1.094094146 | 8.67545E-51 | down |
| ENSCAFG00000034431 | -1.141282248 | 0.000360402 | down |
| ENSCAFG00000049727 | -1.143417406 | 0.021899615 | down |
| ENSCAFG00000031502 | -1.161404377 | 0.005880266 | down |
| ENSCAFG00000041413 | -1.164464107 | 0.015192875 | down |
| ENSCAFG00000035855 | -1.187041395 | 0.005523403 | down |
| ENSCAFG00000042676 | -1.195609663 | 8.87056E-30 | down |
| ENSCAFG00000033034 | -1.227200283 | 1.19974E-05 | down |
| ENSCAFG00000036661 | -1.236292307 | 0.023626022 | down |
| ENSCAFG00000047870 | -1.240357823 | 4.06075E-11 | down |
| ENSCAFG00000041395 | -1.279466806 | 0.026404998 | down |
| ENSCAFG00000043883 | -1.326017776 | 0.046624299 | down |
| ENSCAFG00000048177 | -1.348191174 | 5.45084E-06 | down |
| ENSCAFG00000044050 | -1.38745152 | 0.042728457 | down |
| ENSCAFG00000043172 | -1.400792444 | 0.025074996 | down |
| ENSCAFG00000009420 | -1.422461568 | 3.76543E-14 | down |
| ENSCAFG00000037458 | -1.449695827 | 0.048871339 | down |
| ENSCAFG00000043967 | -1.462987921 | 3.52126E-21 | down |
| ENSCAFG00000048910 | -1.506049 | 6.59534E-13 | down |
| ENSCAFG00000005852 | -1.598597815 | 0.000302809 | down |
| ENSCAFG00000049306 | -1.616524237 | 0.00025275 | down |
| ENSCAFG00000043786 | -1.617647151 | 2.33568E-13 | down |
| ENSCAFG00000039344 | -1.669360757 | 3.05944E-06 | down |
| ENSCAFG00000046278 | -1.723235284 | 0.026599026 | down |
| ENSCAFG00000045536 | -1.736916956 | 1.66101E-24 | down |
| ENSCAFG00000046392 | -1.87203287 | 0.007422414 | down |
| ENSCAFG00000045774 | -1.949274615 | 0.043000768 | down |
| ENSCAFG00000047400 | -1.962843212 | 0.000756732 | down |
| ENSCAFG00000048636 | -1.968093691 | 0.039019816 | down |
| ENSCAFG00000044581 | -2.044431393 | 0.028676184 | down |
| ENSCAFG00000019465 | -2.18044747 | 0.010363133 | down |
| ENSCAFG00000003955 | -2.354864523 | 0.000239792 | down |
| ENSCAFG00000047443 | -2.384162892 | 0.030824075 | down |
| ENSCAFG00000048861 | -2.415977657 | 0.037708458 | down |
| ENSCAFG00000037122 | -2.45496393 | 0.000175574 | down |
| ENSCAFG00000037162 | -2.485952565 | 0.008047432 | down |
| ENSCAFG00000033183 | -2.490908087 | 4.07829E-13 | down |
| ENSCAFG00000049291 | -2.51838641 | 6.07653E-23 | down |
| ENSCAFG00000044765 | -2.678942457 | 8.92017E-07 | down |
| ENSCAFG00000035464 | -2.715127637 | 0.000206906 | down |
| ENSCAFG00000033145 | -2.723451429 | 8.81184E-25 | down |
| ENSCAFG00000033364 | -2.779895841 | 5.6505E-104 | down |
| ENSCAFG00000047031 | -2.834779307 | 9.22817E-06 | down |
| ENSCAFG00000038009 | -3.046074512 | 3.54808E-20 | down |
| ENSCAFG00000009385 | -3.0967157 | 6.05698E-52 | down |
| ENSCAFG00000049023 | -3.157287531 | 0.040336159 | down |
| ENSCAFG00000044319 | -3.182850093 | 3.50344E-05 | down |
| ENSCAFG00000044630 | -3.545764896 | 0.044012176 | down |
| ENSCAFG00000045431 | -3.807308559 | 0.023650778 | down |
| ENSCAFG00000045278 | -3.88612593 | 0.018604552 | down |
| ENSCAFG00000042069 | -4.00993004 | 0.013955197 | down |
| ENSCAFG00000007585 | -4.025848482 | 0.002097162 | down |
| ENSCAFG00000042138 | -4.068226501 | 0.034955198 | down |
| ENSCAFG00000034659 | -4.073522493 | 0.036460945 | down |
| ENSCAFG00000041388 | -4.462132454 | 0.000351896 | down |
| ENSCAFG00000046932 | -4.506668604 | 0.003601012 | down |
| ENSCAFG00000043882 | -4.537255942 | 0.00470333 | down |
| ENSCAFG00000042439 | -4.61699725 | 0.002357728 | down |
| ENSCAFG00000035612 | -4.750018575 | 0.001450649 | down |
| ENSCAFG00000030545 | -5.136133028 | 0.000341986 | down |
| ENSCAFG00000016514 | -5.591853791 | 4.55786E-05 | down |
| ENSCAFG00000049227 | -6.155196645 | 1.19488E-05 | down |
| ENSCAFG00000042065 | -8.209785202 | 8.6332E-11 | down |
