# Supplementary material for: LncRNA-42060 Regulates Tamoxifen Sensitivity and Tumor Development via Regulating the miR-204-5p/SOX4 Axis in Canine Mammary Gland Tumor Cells
Source: Front Vet Sci. 2021 Jun 21;8:654694. doi: 10.3389/fvets.2021.654694 (PMC8255626; doi:10.3389/fvets.2021.654694)
Supplement: Supplementary file 5 [file Table_5.docx]

**Supplementary table 5 Differentially expressed of miRNAs in TAMRs and CMGTs**

| **AccID** | **log2FC** | **FDR** | **Style** |
| --- | --- | --- | --- |
| cfa-miR-204 | -5.789796687 | 6.7966E-19 | down |
| cfa-miR-152 | -3.380094217 | 6.53212E-37 | down |
| cfa-miR-184 | -3.201420675 | 3.67314E-93 | down |
| cfa-miR-218 | -1.984120206 | 1.94501E-70 | down |
| cfa-miR-210 | -1.942356856 | 1.4377E-112 | down |
| cfa-miR-375 | -1.854995417 | 1.3365E-102 | down |
| cfa-miR-146b | -1.690639123 | 1.93533E-36 | down |
| cfa-miR-381 | -1.60870175 | 0.035719806 | down |
| cfa-miR-221 | -1.581200571 | 2.4411E-114 | down |
| cfa-miR-147 | -1.52313882 | 2.67816E-41 | down |
| cfa-miR-222 | -1.425279563 | 7.07483E-92 | down |
| cfa-miR-582 | -1.23246383 | 4.24509E-13 | down |
| cfa-miR-125a | -1.192182684 | 2.50862E-81 | down |
| cfa-let-7e | -1.106810845 | 8.58531E-53 | down |
| cfa-miR-99b | -1.050494303 | 9.72463E-60 | down |
| cfa-miR-8826 | 6.663583168 | 1.42E-09 | up |
| cfa-miR-486 | 2.327248751 | 2.41E-08 | up |
| cfa-miR-133c | 2.205630789 | 1.98E-17 | up |
| cfa-miR-34c | 2.199997492 | 5.33E-58 | up |
| cfa-miR-34b | 2.137597689 | 6.55E-13 | up |
| cfa-miR-1 | 2.116919409 | 2.39E-18 | up |
| cfa-miR-133a | 2.063017983 | 2.66E-14 | up |
| cfa-miR-133b | 1.790758138 | 0.022697642 | up |
| cfa-miR-486-3p | 1.678956144 | 1.85E-05 | up |
| cfa-miR-10b | 1.371348956 | 1.02E-85 | up |
| cfa-miR-181a | 1.195384901 | 5.28E-68 | up |
| cfa-miR-187 | 1.181914543 | 2.39E-35 | up |
| cfa-miR-8903 | 1.07814606 | 0.000372737 | up |
| cfa-miR-2387 | 1.029930739 | 0.002818105 | up |
